# Analytical Differentiation of Wines from Three Terroirs Located in a Warm Winegrowing Area Based on Their Volatilome

**DOI:** 10.3390/molecules30020238

**Published:** 2025-01-09

**Authors:** José Miguel Fuentes-Espinosa, Raquel Muñoz-Castells, Jaime Moreno-García, Teresa García-Martínez, Juan Carlos Mauricio, Juan Moreno

**Affiliations:** Department of Agricultural Chemistry, Edaphology and Microbiology, Agrifood Campus of International Excellence CeiA3, University of Córdoba, 14014 Córdoba, Spain; b42fuesj@uco.es (J.M.F.-E.); raquelmunozcastells@gmail.com (R.M.-C.); b62mogaj@uco.es (J.M.-G.); mi2gamam@uco.es (T.G.-M.); mi1gamaj@uco.es (J.C.M.)

**Keywords:** wine, terroir, volatile compounds, statistical analysis

## Abstract

This research aims to identify aroma compounds, their combinations, and statistical relationships to classify and characterize wines produced in small, defined areas known as “terroirs”, which share edaphoclimatic characteristics grape varieties, viticultural practices, harvest timing, and winemaking processes. The goal is to deepen the understanding of the relationship between the terroir and wine typicity. This study analyzed the contents based on enological parameters, the major and minor volatile compounds of the young wines produced in three wineries across two vintages, using the Pedro Ximenez white grape variety cultivated in different terroirs within the same quality zone. Statistical tools, such as Multiple Variable Analysis (MVA) and Principal Component Analysis (PCA), were employed to identify significant differences in the volatilomes of wines. PCA effectively differentiated wines from each terroir and vintage using scores from the first two principal components, calculated based on the absolute concentrations of 12 major volatile compounds and 3 polyols. Conversely, PCA based on the concentrations of 52 minor volatile compounds showed a strong ability to classify wines by vintage year. The minor volatile contents of wines from 2 vintages, grouped into 9 chemical families, provide distinct fingerprints that enable wines to be distinguished by terroir. The results underscore the chemical basis underlying terroir typicity in a production zone within a warm Protected Denomination of Origin (PDO).

## 1. Introduction

Winemaking processes have traditionally been considered the application of empirical knowledge in chemistry and biology, aimed at favoring the activity of selected microorganisms. However, from this traditional approach to today’s industrial fermentations, winegrowers and winemakers have modified their practices by applying advances in scientific knowledge. As a result, new strategies in oenology are emerging that aim to combine or replace the complexity of spontaneous fermentations with the safety of controlled fermentations with selected yeasts leading to the production of homogeneous wines with the same analytical and sensory characteristics anywhere in the world. On the other hand, wines with their own peculiarities, traditionally produced in certain wine-growing areas of proven quality, are widely accepted by consumers and generate significant economic returns, which has a positive impact on the resilience of the population in these rural areas.

The intricate relationship between the traditional winegrowing areas and the quality of their wines has long captivated the interest of viticulturists, wine makers, and scientists alike. Also, wine consumers often ascribe the unique flavor profiles of their favorite wines to the concept of the terroir, which is currently considered one of the most important concepts in the world of viticulture and oenology. Far from being a rural term, the terroir hides a wide range of analytical parameters that are the basis of the trend of knowledge-based oenology [1]. The terroir embodies the interplay among the natural environment, the vine, and human influence that ultimately shapes the sensory characteristics and quality of the wine. As consumers become more discerning, they are seeking wines that convey a sense of place, underscoring the importance of the terroir in winemaking. The concept of the terroir has been employed for a variety of purposes, including the authentication of products against fraud, the justification of economic advantages associated with a particular property, the synthesis of a historical and local experience, the strengthening of a community of growers facing economic competition, and the explanation of the characteristics or typicity of wines [2].

The terroir is often associated with traditional European wine regions, and it is increasingly recognized worldwide as a key determinant of the wine quality and style. The Organization International of wine [3] define the vitivinicultural terroir as an area in which collective knowledge of the interactions between the identifiable physical and biological environment and applied cultural practices develops, providing distinctive characteristics for the products originating from this area. The terroir includes specific soil, topography, climate, landscape characteristics, and biodiversity features.

While the influence of the grape variety and winemaking techniques cannot be underestimated, it is the interaction of the soil, climate, topography, and human practices that gives a wine its distinctive character, making the terroir the subject of intense scientific studies, which has been summarized in several reviews about the role of the edapho-climatic factors and human practices on the soil properties, composition, and effects on grape composition and quality [4,5,6,7]. Also, the role of microorganisms in the entire winemaking process, mainly their ecological niches, population dynamics, and relationships between “microbiome-vine health” and “microbiome-wine metabolome” should be understood [1,8,9]. Microbial communities respond to environmental changes within a fully established grape-related ecosystem at different scales, where the microbial biogeography of wine grapes is conditioned by the cultivar, vintage, and climate [10,11,12]. The responses to these factors, which drive the distributions of subregional and varietal microbiota, thereby influence the volatile compositions of finished wines and result in regional wine typicity based on volatile profiles [13]. In this context, several studies have focused on the composition of volatile compounds to distinguish wines obtained from the same grape variety grown in different regions or from different grape varieties cultivated in the same region. The secondary metabolites of grapes—such as terpenes, benzene derivatives, and ketones—along with fermentation byproducts, like esters and alcohols, have been identified as key factors influencing regional and varietal differences in wines [14,15,16,17,18,19,20,21]. However, there is a notable lack of research dedicated to the objective characterization or typicity of the traditional wines made from the same grape variety in specific terroirs within the same PDO.

A recent study assessed the influence of soil properties on grape production in six terroirs from three different production areas, two premium-grade and one standard-grade area, with the aim of applying this concept to the zonification of the Montilla-Moriles PDO wine-growing area (Andalusia, southern Spain) [22]. This DOP is widely recognized for the high quality of its traditional wines produced from the white grape variety Pedro Ximénez.

In order to deepen the relationship between terroir and wine typicity, the instrumental analysis of their volatilome provides a large number of variables and data, so it is appropriate to use a multivariate analysis statistical approach, such as Principal Components Analysis (PCA). This chemometric tool allows for the exploration of data without prior assumptions (e.g., predefined group labels), while reducing their dimensionality.

The primary aim of this work is to extract relevant, objective, and useful information to help establish a criterion for the typification of wine products from a specific terroir. To achieve this goal, this study includes a quantitative analysis of the main oenological variables, the major volatile and the minor volatile compounds in wines made from the Pedro Ximénez white grape cultivated in three specific terroirs and two consecutive vintages.

## 2. Results and Discussion

To objectively evaluate the effects of the terroir, vintage, and their interaction on each of the quantified compounds, a two-way MANOVA was performed. This statistical method provides information about the significance levels of these two factors and their interaction.

The results indicate that nearly all compounds are significantly affected (*p* ≤ 0.05) by both factors and their interaction. Only a few compounds are not significantly influenced by these factors or their interaction. These compounds are listed in Table S1 of the Supplementary Material. According to the table, among the general parameters, only pH, total acidity, and absorbance at 620 nm are significantly influenced by the terroir, while reducing sugars are affected by the vintage. All of these parameters are also influenced by the interaction, with the exception of density. Among the 15 minor volatiles and polyols quantified, only 2-methyl-1-butanol shows no dependence on the interaction, and acetoin is not affected by the vintage. Lastly, of the 52 minor volatiles, only 8 are not influenced by one or more of the three factors considered, while the remaining 47 volatiles are significantly dependent on the terroir, vintage, and their interaction.

### 2.1. Main Oenological Variables for Wine Characterization

The bunches of Pedro Ximénez grape grown in the three terroirs studied are subjected traditionally to a partial dehydration on the vine in order to increase their sugar content to around 255 g L^−1^. The must obtained from these grapes allows wines with a 15% *v*/*v* ethanol content to be obtained in a natural way. These wines, after about one year of conservation in stainless steel tanks or cement vats, are subjected to a biological aging process in oak barrels under a flor veil of *S. cerevisiae* yeasts, characteristic of the traditional Andalusian “fino” type wines.

The means, deviations, and homogeneous groups (HGs) obtained for each of the 12 variables tested in the young wines, are listed in Table 1. Among them, only the total acidity and the absorbances at 280, 420, and 520 nm show 6 HG in agreement with the six wine samples, revealing their dependence on the terroir and vintage. Also, five variables (ethanol, volatile acidity, malic and lactic acid, and absorbance at 620 nm) show five HGs, suggesting a relationship between their values and the vintage or terroir for some of the three wineries tested. The variables pH, reducing sugars, and density show four, three, and two HGs, respectively. These values correspond to a complete transformation of sugars in ethanol and therefore the production of dry wines in all vintages and wineries.

The ethanol content, volatile acidity, total acidity, and pH have values within the range of the wines produced in the terroirs under study and do not show significant variations from one winery to another. Only the 2021 wines from the SA and AG wineries have higher volatile acidity values than the other wines. This is probably due to a low level of contamination of the musts with undesirable microorganisms from the rotten or damaged grapes or to the presence of non-*Saccharomyces* yeasts from the specific terroir [10,12]. Regarding this, it has been proven that *Saccharomyces cerevisiae* fermentative yeasts do not usually produce volatile acidity contents higher than 0.3 gL^−1^ in white wines from this wine-growing PDO [23,24,25]. The total acidity and pH values are mainly due to pre-fermentative or finishing treatments with the addition of tartaric acid [26] necessary in this warm area. Their values show scarce variation between wineries and vintages. In addition, fertilization treatments with potassium salts and the water regime of the vineyard in each year influence the acidity of the must [27] and therefore the need to adjust it to the traditional levels of this area (4 to 5 gL^−1^).

The malic acid comes from the grape and its content in wines, and together with the lactic acid content, is mainly determined by the malolactic fermentation carried out by malolactic bacteria. This fermentation is desirable for red wines, but not for young white wines, because it provides fresh, acidic notes that are pleasant to the palate. The values in Table 1 show the significant influence of the terroir in the wines of SA2021 and SA2022, as these wines have the highest malic acid content. In this regard, some authors [22] found similar results in their study of these terroirs of this PDO and relate them to the characteristics of the red soils abundant in the area surrounding this winery. Only wine LU2022 shows 0.02 g L^−1^ of malic acid and 0.97 g L^−1^ of lactic acid, probably as a consequence of spontaneous malolactic fermentation, because although some *Saccharomyces cerevisiae* yeast strains metabolize malic to lactic acid during the alcoholic fermentation, their production does not exceed 0.4 g L^−1^ [28].

The reducing sugar contents in wines include the non-fermentable sugars and are regulated by international standards and the regulatory bodies of the specific wine-growing area. They are closely related to the density values measured after fermentation is completed or terminated and vary widely, depending on the wine type. Thus, dry wines have sugar contents (glucose + fructose) and density values lower than 4 g L^−1^ and 995 g L^−1^, respectively, and the sweet wines can have contents higher than 45 g L^−1^ [29] with density values higher than 1000 g L^−1^. The values shown in Table 1 indicate that fermentation has been completed in all wineries and that the wines obtained are dry wines.

Absorbance measurements in the visible light spectrum at 420, 520, and 620 nm are due to the presence of phenolic compounds and are used by winemakers to establish color indices (color intensity and hue) in both white and red wines, while the absorbance measurement at 280 nm is an index of the total polyphenol content of the wine. The composition and content of different polyphenol families, including these fractions, contribute to the color, flavor, oxidative stability, astringency, and bitterness of wines [30]. The values of absorbance at 280, 420, and 520 nm (Table 1) show six HGs, indicating their relationship with the terroir and vintage. Nevertheless, the absorbance at 620 nm shows five HGs with similar values in the 2021 vintage wines from the SA winery and in the 2022 vintage wines from the AG and LU wineries.

### 2.2. Major Volatile Compounds

Volatile organic compounds (VOCs) are responsible for the wine aroma. Which ones are present and in what amounts depends on variables related to the terroir and, in the last instance, to the yeast growing in the winemaking process [31].

Table 2 shows the contents in the major VOCs and polyols quantified in this work. This group of VOCs consists of methanol and the higher alcohols, carbonyl compounds, and ethyl esters with a low molecular weight, all of which have a content greater than 10 mg L^−1^. The content of polyols 2,3-butanediol (*meso* and *levo* isomers) and glycerol are also shown. All of these compounds come from the metabolism of yeasts during alcoholic fermentation, with the exception of methanol, which comes from the enzymatic hydrolysis of grape pectins [32].

The higher alcohols isobutanol and propanol, exhibit six and five HGs respectively, while the remaining alcohols show four HGs. Carbonyl compounds, mainly acetaldehyde, were found in larger concentrations in wines from the 2021 vintage in three wineries, showing five HGs. This aldehyde is formed to levels around 100 mgL^−1^ during the growth of fermentative yeasts. The higher amounts in the wines of this year should be explained by the presence of non-*Saccharomyces* yeast in the grape-must of this vintage and mainly by the presence of the *S. cerevisiae* flor yeasts after the fermentative process. These yeasts are characteristic of this production area [33]. Acetaldehyde serves as a precursor metabolite for the synthesis of acetate, acetoin, and other compounds. The 1,1-diethoxyethane is a characteristic compound of wines biologically aged under flor veil and is a product of the reaction of acetaldehyde with ethanol in wines with a high content of both compounds. Its content in LU2021 wines is related to an initial stage of this process. The ethyl esters of acetic acid and succinic acid show four HGs, while ethyl lactate shows five. Lastly, the contents in the isomers of 2,3 butanediol exhibit four HGs, with glycerol having only two HGs.

The application of conventional statistical methods to analyze the influence of the terroir and/or vintage on all the variables quantified in complex samples as wine does not prove to be very efficient to show the similarity of wines produced in the three terroirs and two vintages under study. In such cases, interpreting experimental data becomes more straightforward with the use of multivariate analysis (MVA) techniques, such as Principal Component Analysis (PCA). This treatment is highly objective, because it relies on mathematical transformations to maximize variance and reduce dimensionality without requiring assumptions about the underlying data distribution or relationships. PCA is independent of predefined groups or assumptions about latent variables, and its results are entirely driven by the data and its variance structure. It identifies patterns in multidimensional data matrices, allowing for the selection of fewer results with comparable characteristics.

To study the influence of the terroir within the same production area and the vintage year on the compositions of wines, a PCA was performed on the data matrix of the major volatiles and polyols quantified in all the wines. This analysis visualizes potential sample clustering. PCA extracts a reduced number of principal components, defined as a set of linear orthogonal combinations that explain the greatest variance of the information content in a larger set of quantitative variables. This method provides an initial approach to explore the classification of wines using a limited set of variables that can be quantified in a single step through an easy-to-use technique, as is the GC methodology employed in this work.

**Figure 1:**
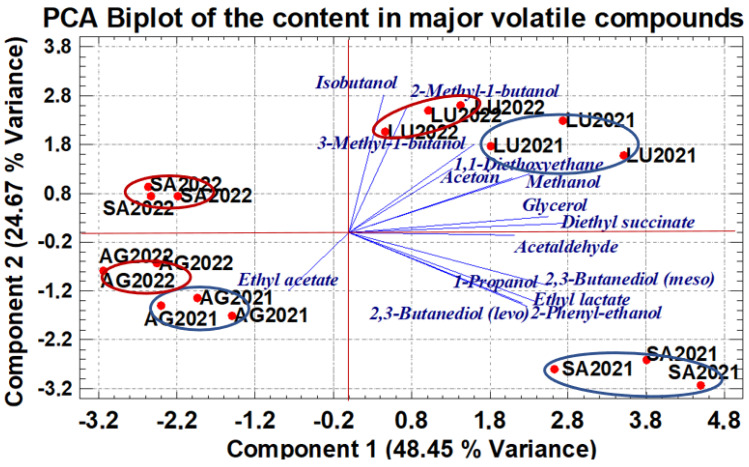
Principal component analysis performed with the data matrix of the major volatile compounds quantified in wines. The red bold circles represent the wines of terroirs and their coordinates, their scores in each PC. The projection of the end of each line on the axis is proportional to its contribution of the PCs.

The result obtained is shows in Figure 1 by means of a biplot using, as axes, the values of the PC1 and PC2, which together explain 73.12% of the total variance. This biplot shows a clear differentiation of the wines according to their terroir for each vintage. A grouping of the wines from the two vintages considered is also obtained for the AG and LU indicating a similar vintage effect in these terroirs. In contrast, the vintage effect is larger for the SA winery, as the 2021 wines are in the lower right quadrant of the biplot, while the 2022 wines are in the upper left quadrant.

The PC1 explains 48.45% of the total variance and can be related to the terroir effect, since its scores group the wines along the X-axis by winery. This component 1 is strongly influenced by nine variables with a high positive load (Table S2 of Supplementary Material). They are methanol (0.299), 2-phenylethanol (0.287), acetaldehyde (0.274), acetoin (0.270), ethyl lactate (0.309), diethyl succinate (0.355), 2,3-butanediol (*levo* and *meso*) (0.296, 0.329), and glycerol (0.330). Component 2, which explains 24.67 of the variance, is mainly driven by the higher alcohols (1-propanol, isobutanol, 2-methylbutanol, and 3-methylbutanol, which have loads higher than 0.314 (Table S2 Supplementary Material). All of them play a key role in the separation of wine samples according to vintage.

### 2.3. Minor Volatile Compounds

These volatiles are found in wine at concentrations of less than 10 mg L^−1^. Despite their low levels, they usually have a great influence on the wine aroma due to their low olfactory perception thresholds. Table 3 shows the contents of the 52 minor volatile compounds quantified. They are grouped into nine chemical families: eight acetates of higher alcohols, twelve ethyl esters of organic acids with a short, medium, and long carbon chain, five other esters, six higher alcohols, two volatile phenols, four lactones, eight carbonyl compounds, six terpenes, and one miscellaneous compound.

Esters are the largest family of volatile compounds in wine. They are metabolites produced by yeasts during alcoholic fermentation and give a pleasant fruity aroma to the wine. Among the 25 quantified esters, only 3, 2-phenyl-ethyl acetate, ethyl octanoate, and ethyl decanoate, have 6 HGs that significantly differentiate the 6 wines by terroir and vintage. Five esters (isoamyl acetate, geranyl acetate, ethyl dodecanoate, ethyl tetradecanoate and ethyl decanoate) have 5 HGs, and 7 esters show 4 HGs. Most acetates (with the exception of n-octyl acetate) and certain ethyl esters had substantially greater values in wines from 2022 than in 2021. This is probably due to uncontrolled factors typical of industrial fermentations, such as the development of non-*Saccharomyces* yeast species in the early stages of fermentation and/or the response of the fermentative *Saccharomyces* yeasts to stress conditions. Among the higher alcohols, only decanol shows six HGs, octanol and furanmethanol 4, and the remaining alcohols show three HGs.

The contents of volatile phenols (4-ethylguaiacol and 2-methoxy-4-vinyl-phenol) are due to the grape [15] or to the activity of non-*Saccharomyces* yeast, also coming from the grape [13]. Levels of this family in wines has been found to be significantly dependent on the region of wine production [34]. They are considered off-flavors with metallic, meaty, or putrid aromas [35]. Only 2-methoxy-4-vinyl-phenol has a quantifiable level in this study, showing six HGs.

Within the lactone family, γ-butyrolactone, which is produced by the yeast *S. cerevisiae* [36], along the fermentation process, has the highest content and shows only two HGs. In contrast, β-lactone and nonalactone have six and five HGs, respectively. This family is recognized as potent odorants in wines and their contents are related to their precursors in grapes and the wine aging in oak barrels [37]

The group of minority carbonyl compounds consists mainly of aldehydes with six, eight, and ten carbon atoms. They are derived from the enzymatic hydrolysis of long-chain fatty acids. Octanal, with two HGs, and decanal and hexanal, with four HGs, are the most representative of this family. Furfural or furaldehyde also has four HGs, and their contents in wines can be the result of their formation from the sugars in the grapes that are partially dehydrated on the vine, as is traditional in this warm production zone where high temperatures are reached during the ripening and harvesting period.

The Pedro Ximénez grape, from which all the wines in this study were obtained, is classified as a neutral variety because its terpene content is below 0.3 mg L^−1^, which results in a weak contribution to the wine aroma [38]. All terpenes and their derivatives quantified in this study have concentrations below this range, and only the sum of limonene isomers is above in the SA winery and in the 2022 vintage. E-citral and geranyl acetone are the only compounds in this family with a higher number of homogeneous groups, with five and four HGs respectively.

Lastly, 2-pentylfuran is the only component of the miscellaneous set. This compound has been described as a potent odorant in bread and as a product of Maillard reactions [39]. It has an odor descriptor of fruity, green, earthy beany with vegetable-like nuances, and its presence in wines from this area can be explained by the high temperatures reached during the ripening period and by partial dehydration of the grapes, as in the case of furaldehyde.

**Figure 2:**
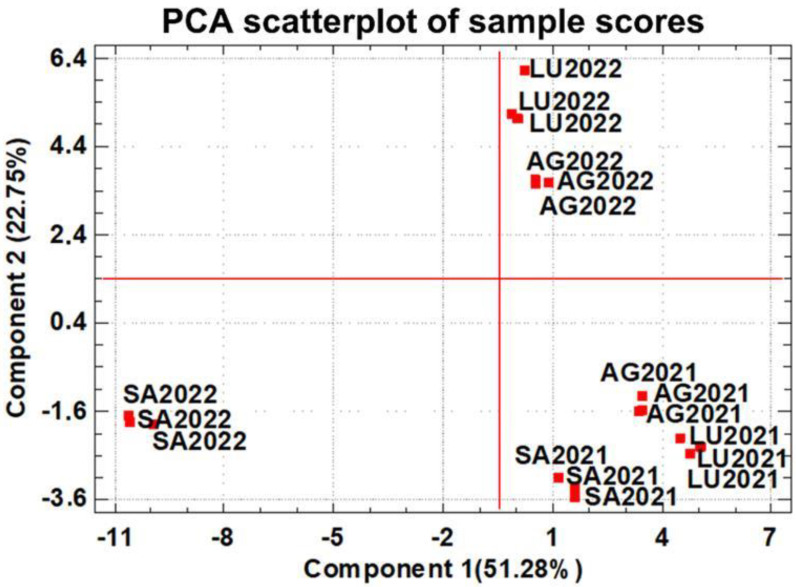
Principal Component Analysis performed with the data matrix of the 52 minor volatile compounds quantified in wines. The red bold squares represent the wines of terroirs and their coordinates of their scores in each PC. Loads of the minor volatile compounds on the PCs are shown in Table S2, Supplementary Material.

The data matrix built with the concentrations of the 52 minor volatile compounds was subjected to Principal Component Analysis (PCA), and the results are shown in Figure 2 and Table S3 in the Supplementary Material. The first two components accounted for 74.03% of the total variance (51.28% for PC1 and 22.75% for PC2). The scores of the wine samples in both PCs are plotted in Figure 2, and the contribution (loadings) of each compound to the PCs are listed in Table S3; these loadings allow for the identification of the most important compounds influencing each PC. Figure 2 shows how the wines are grouped by vintage year. All wines from vintage 2021 are located in the lower right corner of the plot, indicating a strong similarity among them. This distribution is mainly driven by the influence of compounds with positive loadings in PC1, including 2-ethyl-1-hexanol, ethyl isobutyrate, γ-nonalactone, and γ-butyrolactone, as well as negative loadings in PC2, such as ethyl 2-methylbutanoate, ethyl 3-methylbutanoate, and ethyl heptanoate. In contrast, the 2022 wines are shifted towards the upper part of the plot along PC2, which is mainly influenced by compounds with positive loadings, such as 3-heptanone, butyl acetate, hexanol, octanal, geranyl acetate, farnesol, and phenethyl benzoate.

Samples from the 2022 vintage and SA winery (SA2022) are highlighted as the most different and they are grouped in the lower left quadrant, with negative scores in PC1 and PC2. The strong influence of compounds, such as ethyl butanoate, ethyl hexanoate, ethyl octanoate, ethyl decanoate, ethyl undecanoate, ethyl tetradecanoate, pentylfuran, limonene, E-2-octenal, 2-methoxy-4-vinylphenol, β-damascenone, hexyl hexanoate, and phenethyl butyrate, all of which have negative loadings in PC1, explain this effect.

Figure 2 shows that Component 2 groups wine samples by vintage and Component 1 differentiates the wines by terroir in a less clear way. This fact is probably due to the anomalous 2022 vintage at the SA winery and shows the need to deepen these investigations by analyzing more vintages, mainly to avoid the impact of anomalous grape ripening due to adverse climatic conditions or grape diseases.

### 2.4. Tentative Fingerprinting to Each Terroir Based on Volatile Compounds

**Figure 3:**
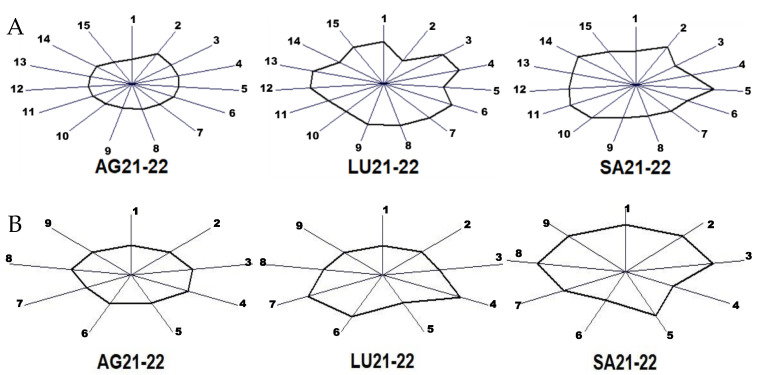
Fingerprint obtained via a multiple variable analysis of the average contents of wines from two vintages in major volatile compounds (**A**) and minor volatile compounds grouped in the nine chemical families (**B**). Major volatiles in (**A**): 1, acetaldehyde; 2, ethyl acetate; 3, 1,1-diethoxiethane; 4, methanol; 5, 1-propanol; 6, isobutanol; 7, 2-methyl-1-butanol; 8, 3-methyl-1-butanol; 9, acetoin; 10, ethyl lactate; 11, 2,3-butanediol (*levo*); 12, 2,3-butanediol (*meso*); 13, diethyl succinate; 14, 2-phenylethanol; 15, glycerol. Chemical families in (**B**): 1, acetates; 2, ethyl esters; 3, other esters; 4, higher alcohols; 5, phenols; 6, lactones; 7, carbonyl compounds; 8, terpenes and derivatives; 9, miscellaneous. Each sunray shows the value for each variable scaled from the mean plus 3 times its deviation at the extreme and minus 3 times its deviation at the origin.

Finally, the sum of the contents of major and minor volatile compounds obtained for the wines of the two vintages was subjected to an MVA in order to easily visualize the in-fluence of the terroir by means of a fingerprint. The results are presented in Figure 3.

The fingerprints are obtained using the so-called glyph or sunray plot, which are multivariate visualization techniques useful to identify differences and similarities among observed cases when the number of dimensions is too large. The glyph is a polygon that represents the values of each quantitative variable. The size of the polygon in each direc-tion is scaled according to the value of each variable for the winery associated with one terroir. Wineries with similar characteristics will have a similar size and shape. The origin of the rays represents the mean content of each variable minus 3 times its deviation and the extreme corresponds to the mean plus 3 times the deviation. This is because the mean and sigma scale was selected for the MVA.

Figure 3A shows that the wines from the two vintages of winery AG have the most regular polygon and winery LU the most irregular, with the higher contents in 1,1-diethoxiethane and methanol. Winery SA has the higher levels of ethyl acetate and propanol. Regarding the chemical families of the minor volatiles, winery AG has the most regular glyph considering the two vintages. LU shows higher levels in higher alcohols and carbonyl compounds, and SA has the highest values in all the families except for the higher alcohols and lactones.

Variations in wine volatile composition across vintages within the same terroir can be attributed to the pronounced geographical heterogeneity of microbiota, which shapes the distribution of subregional and varietal communities and, in turn, influences the wine’s volatile profile [13].

In this context, some authors propose that the vintage (the year of isolation) plays a more important role than the terroir (geographical location) in determining yeast biodiversity in grapes [37,40], This finding helps to explain the differences observed in wines from vintages 2021 and 2022 at the SA winery. However, results from other wineries in this study show similar values across the two years, suggesting that the terroir exerts a strong influence when its microclimatic conditions are favorable.

The findings of this study demonstrate that the analysis of wine volatile compounds (major and minor), leveraging advanced statistical tools, such as PCA, can effectively authenticate wines as representative products of specific terroirs. Further research is essential to deepen our understanding of the complex relationship between the terroir and wine quality. Such studies should aim at establishing objective markers based on key volatile compounds to ensure the typicality and quality of wines from traditional production areas.

## 3. Materials and Methods

### 3.1. Brief Description of the Winegrowing Area and Terroir

A recent study describes different production areas within the Protected Denomination of Origin (PDO) of Montilla-Moriles, ubicated in the province of Córdoba (Andalusia, southern of Spain) according the soil properties and the wines obtained in connection with the “terroir” [22]. Two high-quality or premium-grade winegrowing areas are located in the mountains near of the towns, Montilla (Sierra de Montilla) and Moriles (Moriles Altos). These areas have undulating lands with soils called “albarizas”. Another third zone or standard-grade area is called “Ruedos”, with soils developed on varied materials, including Miocene marls, which are steeply and highly eroded. The Ruedos vineyards are more closely related to the soil sand and available Mn and also to scarcely available P and Zn [22]. The Pedro Ximénez grape is the main variety cultivated in this PDO, and it is strictly recommended that the soluble solid content of grapes in the harvesting period reach 24.7–25.4 ºBrix [41], equivalent to 250–257 g L^−1^. This sugar content is reached through the partial dehydration of the grape bunches on the vine for about seven-dix days and allows the traditional “Fino” type wine to be obtained, with an ethanol content of about 15% *v*/*v*. The grape harvest in this area takes place from late August to mid-September, when grapes reach the industrial degree of ripeness suitable for the type of wine to be obtained.

### 3.2. Wineries, Fermentation Technology, and Wine Samples

Three wineries, La Unión (LU), Aguilar (AG), and San Acacio (SA), were selected in the areas corresponding to the Montilla, Aguilar de la Frontera, and Montemayor towns, respectively, which are recognized as representative cellars of the “Ruedos” Zone. These wineries produce their wines using the traditional winemaking techniques and only the grapes grown in their immediate surroundings and are located at the following coordinates: LU, 37°34′36″ N, 4°38′08″ W; A, 37°30′37″ N, 4°39′11″ W; and SA, 37°38′22″ N, 4°42′04″ W; within the PDO Montilla-Moriles. A recent edapho-climatic study of this grape-growing zone [22] describe their soils as developed on varied materials, including Miocene marls, which are steeply and highly eroded. The vineyards from the Ruedos are more closely related to the soil sand and available Mn and also to the scarcely available P and Zn; however, grape bunches were more abundant and grapes were larger (100-berry weight), and their corresponding musts are richer in malic acid [22]. In this regard, the technology of vinification to obtain white wines in warm regions does not generally include the malolactic fermentation, which is always desirable for red wines. In addition, the malic acid content in the musts of grapes grown in these regions is rarely higher than 1 gL^−1^. The yeasts used for the production of each wine were those from the autochthonous microbiota naturally present on the grape skins and must, which vary across different terroirs. This microbiota underwent a process known as “pied de cuve” a traditional winemaking practice used to select fermentative *S. cerevisiae* strains and acclimate them to fermentation conditions at larger scales. In addition, portions of fresh must are added to the fermentation tanks, when the yeasts are in their exponential growth phase, thus ensuring complete fermentation and the production of dry white wines on an industrial scale. This fed-batch winemaking technology merges the benefits of batch and continuous fermentation. It begins like batch fermentation, but fresh musts are added intermittently as the process continues. This approach increases productivity by extending the yeasts’ exponential growth phase and preventing starvation, while also mitigating product inhibition by maintaining low concentrations of the final product. Consequently, the fermentation duration extends to about one month for all wines in this work.

For this study, three samples of young wines made from the Pedro Ximénez grape variety were collected from each of the three wineries over two consecutive vintages (2021 and 2022), following a spontaneous stabilization period of approximately one month.

### 3.3. Analytical Methods

The quantification of twelve oenological parameters used for the general characterization of wines was performed using the methods recommended by the OIV [42]. An Agilent Cary 60 UV-Vis spectrophotometer (Agilent technologies, Santa Clara, CA, USA) was used for the determination of the absorbance values at 420, 520, and 620 nm and at 280 nm to obtain the Total Polyphenol Index (TPI). Lactic and malic acids were quantified using a reflectometric method (Reflectoquant™; Merck^®^, Darmstadt, Germany).

The major volatile compounds and polyols in wines were quantified using a Gas-Chromatograph Agilent 6890 plus (Agilent technologies, Santa Clara, CA, USA) equipped with a Flame Ionization Detector (FID) and a fused silica capillary column CP-WAX 57 CB (60 m × 0.25 mm i.d. × 0.4 µm film thickness) by using the method published in a previous paper [23,24,25]. Briefly, this method consists of a previous treatment of 10 mL of the wine sample by adding 1 mL of the internal standard solution prepared with 1018 mg L^−1^ of 4-methyl-2-pentanol (CAS number 108-11-2) in ethanol at 14% *v*/*v*, followed by the addition of 0.2 g of solid calcium carbonate. This mixture was then stirred for 30 s in an ultrasonic bath, followed by a centrifugation at 5000 rpm for 10 min at a 2 °C temperature. After, the liquid phase was transferred to a falcon tube and a volume of 0.7 µL and injected into the GC inlet. The quantification of methanol, higher alcohols (1-propanol, isobutanol, 2 and 3-methyl-1-butanol, and 2-phenylethanol), acetaldehyde, acetoin, ethyl acetate, and the polyols glycerin and 2,3-butanodiol (*levo* and *meso* forms) was carried out based on the calibration curve previously obtained by subjecting standard solutions of each compound to the same treatment as the samples.

Minor volatiles were analyzed using a Stir Bar Sorptive Extraction-Thermal Desorption-Gas Chromatography-Mass Spectrometry (SBSE-TD-GC-MS) analytical platform consisting of an Agilent 7890A GC coupled to an MSD 5975C (Wilmington, DE, USA) and a Gerstel Multi-Purpose Sampler (MPS) (GmbH & Co. KG–Mülheim an der Rhur, Germany). Chemstation (Agilent, version B.04.03) and Maestro (Gerstel, version 1.3) software were used to control the platform conditions and chromatographic data processing. The methodology and regression curves for absolute quantification are described in previous work [43,44,45]. Briefly, the minor volatiles were extracted using the SBSE technique, using a twister (10 mm long, 0.5 mm-thick film) coated with polydimethylsiloxane (PDMS). In this way, 1 mL of the wine sample, 0.1 mL of the internal standard solution (0.4116 g L^−1^ hexyl butyrate in absolute ethanol), and 8.9 mL of a solution containing 2.6 g L^−1^ tartaric acid and 2.2 g L^−1^ potassium bitartrate in ethanol, at 12% *v*/*v* and pH 3.5, were added to a 10 mL vial. The twister was then added to the vial, which was placed in a Variomag Multipoint magnetic stirrer (Thermo Fisher Scientific, Waltham, MA, USA) and stirred at 1200 rpm and 20 °C for 120 min. After this time, the twister was removed, rinsed with water, dried with a soft tissue, and placed in the MPS for transport to the Thermal Desorption Unit (TDU) for the desorption of volatiles and transfer to the GC system. A HP-5MS fused silica capillary column (60 m × 0.25 mm i.d.; 0.25 μm film) from Agilent Technologies was used, and the temperature program was as follows: initial 50 °C (2 min) increasing at 4 °C/min to 190 °C for 10 min. The MSD was operated at 70 eV in electron impact mode (EI) with a mass range of 35-550 Da at a temperature of 150 °C.

All samples of this work were analyzed in triplicate, and all major and minor volatiles were identified and confirmed via GC-MS on the same Agilent 7890-MSD 5975C (Wilmington, DE, USA) previously described with the same conditions used for their analysis. Compound identification was performed by comparing the peak data of the compounds with the mass spectra libraries NIST08 and Wiley7 and by consulting the NIST database from the Web of Chemistry and subsequently based on the linear retention index (LRI) of each compound, according to previous work [43,44,45]. Another identification was performed by subjecting a mixture of commercially available pure compounds to the same analytical conditions as the samples. Reagents and pure chemical compounds for identification and quantification were provided by Sigma-Aldrich (St. Louis, MO, USA) and Merck (Darmstadt, Germany).

### 3.4. Statistical Analysis

Data matrices of the general oenological parameters, major and minor volatile compounds, were subjected to statistical analysis using the Statgraphics Centurion (v. 16.1.11) statistical package. All the quantified compounds were subjected to a Multiple Analysis of Variance (MANOVA) considering the terroir, vendange, and their interactions as variation factors. ANOVA of multiple ranges and F-tests were used for the establishment of homogenous groups. Multiple Variable Analysis (MVA) was used to identify significant differences between the wines from the three terroirs and two vintages. A PCA was also performed to provide a general interpretation of the main information provided by the contents in volatile compounds and polyols quantified and also in the minor volatile compounds.

## 4. Conclusions

Young wines from two consecutive vintages of the white grape variety Pedro Ximénez, produced in three terroirs within the same production zone, were subjected to an analysis of their volatiles and statistical evaluation aimed at establishing their relationship.

Among the general wine parameters, only total acidity and absorbances at 280, 420, and 520 nm showed a significant dependence on both the vintage and terroir.

A Principal Component Analysis (PCA) of the main volatile compounds and polyols identified nine volatiles influencing the grouping of wines by terroir. In addition, three higher alcohols were found to be the most important contributors to the differentiation of wines by vintage.

The PCA performed on the data matrix of the 52 quantified minor volatiles showed a clear grouping of wines by vintage, but not by terroir.

The multivariate analysis performed on the sum of the contents of the major or minor volatile compounds of the wines of the two vintages allows a simple visualization of the terroir influence to be obtained through a fingerprint.

These results shed light on the effects of terroir within the same winegrowing zone on the composition in wine volatiles, highlighting their importance for the wine composition and opening objective procedures to defend the typicality and quality of their products. The need for further research into the complex relationship between the terroir and wine typicity, mainly aimed at establishing objective fingerprints or ranges for the contents of key compounds, is also established.

## Figures and Tables

**Table 1:** Mean values and standard deviations of the oenological parameters of wines from three terroirs in 2021 and 2022 vintages. ^a–f^ Different letters in the same row indicate homogeneous groups with statistical differences at the 0.05 significance level. Identification of wine samples from 2021 and 2022. Aguilar AG2021, AG2022; La Union LU2021, LU2022 and San Acacio SA2021, SA2022.

Oenological Parameters	AG2021	AG2022	LU2021	LU2022	SA2021	SA2022	HGs
Ethanol (% *v*/*v*)	15.4 ± 0.1 ^e^	14.8 ± 0.1 ^c^	15.1 ± 0.1 ^d^	15.1 ± 0.1 ^d^	14.5 ± 0.1 ^b^	14.0 ± 0.1 ^a^	5
pH	3.40 ± 0.00 ^b^	3.48 ± 0.00 ^d^	3.40 ± 0.02 ^b^	3.35 ± 0.00 ^a^	3.49 ± 0.01 ^d^	3.45 ± 0.01 ^c^	4
Volatile acidity (g L^−1^)	0.38 ± 0.02 ^c^	0.50 ± 0.02 ^d^	0.39 ± 0.00 ^c^	0.27 ± 0.00 ^a^	0.57 ± 0.00 ^e^	0.35 ± 0.02 ^b^	5
Total acidity (g L^−1^)	4.73 ± 0.00 ^d^	5.45 ± 0.00 ^f^	4.24 ± 0.00 ^c^	4.09 ± 0.00 ^a^	4.75 ± 0.01 ^e^	4.16 ± 0.00 ^b^	6
Malic Acid (g L^−1^)	0.8 ± 0.1 ^c^	0.66 ± 0.07 ^b^	0.80 ± 0.04 ^c^	0.02 ± 0.00 ^a^	0.87 ± 0.01 ^d^	1.36 ± 0.02 ^e^	5
Lactic Acid (g L^−1^)	0.3 ± 0.0 ^a^	0.39 ± 0.02 ^bc^	0.43 ± 0.01 ^c^	0.97 ± 0.08 ^e^	0.49 ± 0.01 ^d^	0.33 ± 0.03 ^ab^	5
Density (g L^−1^)	986 ± 0 ^b^	986 ± 0 ^b^	985 ± 0 ^a^	985 ± 0 ^a^	986 ± 0 ^b^	986 ± 0 ^b^	2
Reducing sugars (g L^−1^)	0.7 ± 0.0 ^b^	0.43 ± 0.0 ^a^	0.7 ± 0.0 ^b^	0.7 ± 0.0 ^b^	0.96 ± 0.00 ^c^	0.43 ± 0.00 ^a^	3
IPT (Absorbance 280 nm)	7.40 ± 0.02 ^c^	10.19 ± 0.01 ^f^	5.64 ± 0.03 ^a^	8.60 ± 0.01 ^e^	7.71 ± 0.04 ^d^	6.70 ± 0.01 ^b^	6
Absorbance 420 nm	0.177 ± 0.001 ^c^	0.1869 ± 0.0004 ^e^	0.081 ± 0.002 ^a^	0.1901 ± 0.0004 ^f^	0.1841 ± 0.0002 ^d^	0.105 ± 0.001 ^b^	6
Absorbance 520 nm	0.043 ± 0.001 ^e^	0.0387 ± 0.0006 ^c^	0.0195 ± 0.0004 ^a^	0.0409 ± 0.0007 ^d^	0.0439 ± 0.0009 ^f^	0.022 ± 0.001 ^b^	6
Absorbance 620 nm	0.0195 ± 0.0008 ^e^	0.0158 ± 0.0002 ^c^	0.010 ± 0.001 ^a^	0.0180 ± 0.0001 ^d^	0.0168 ± 0.0004 ^cd^	0.012 ± 0.001 ^b^	5

**Table 2:** Major volatile compounds and polyols quantified in wines from three terroirs in 2021 and 2022 vintages. ^a–f^ Different letters in the same row indicate homogeneous groups (HG) with statistical differences at the 0.05 significance level. Identification of wine samples: Aguilar AG2021, AG2022: La Unión LU2021, LU2022: San Acacio SA2021, SA2022. CAS: Chemical Abstracts Service Registry Number.

Compounds (mg L^−1^)	CAS	AG2021	AG2022	LU2021	LU2022	SA2021	SA2022	HGs
Methanol	67-56-1	63 ± 4 ^a^	67 ± 4 ^a^	110 ± 6 ^c^	83 ± 7 ^b^	84 ± 8 ^b^	65 ± 4 ^a^	3
1-Propanol	71-23-8	26 ± 1 ^a^	38 ± 2 ^c^	32 ± 2 ^b^	44 ± 3 ^d^	63 ± 3 ^e^	29.9 ± 0.3 ^b^	5
Isobutanol	78-83-1	16 ± 1 ^a^	26.4 ± 0.9 ^c^	40 ± 2 ^e^	44 ± 1 ^f^	20 ± 1 ^b^	37.1 ± 0.8 ^d^	6
2-Methyl-1-Butanol	137-32-6	29 ± 1 ^a^	34 ± 1 ^b^	43 ± 3 ^c^	52 ± 2 ^d^	34 ± 1 ^b^	42.6 ± 0.6 ^c^	4
3-Methyl-1-Butanol	123-51-3	227 ± 3 ^a^	249 ± 5 ^b^	286 ± 15 ^c^	344 ± 15 ^d^	277 ± 1 ^c^	251.2 ± 0.9 ^b^	4
∑ Isoamyl Alcohols		256 ± 3 ^a^	283 ± 6 ^b^	329 ± 17 ^d^	396 ± 16 ^e^	311 ± 2 ^c^	293.8 ± 0.3 ^bc^	5
2-Phenylethanol	60-12-8	36 ± 4 ^b^	25 ± 1 ^a^	41 ± 3 ^c^	30.8 ± 0.7 ^b^	57 ± 8 ^d^	36 ± 3 ^b^	4
Acetaldehyde	75-07-0	186 ± 10 ^c^	88 ± 4 ^a^	332 ± 28 ^e^	118 ± 3 ^b^	241 ± 18 ^d^	125 ± 5 ^b^	5
1,1-Diethoxyethane	105-57-7	0 ^a^	0 ^a^	2.3 ± 0.2 ^b^	0 ^a^	0 ^a^	0 ^a^	2
Acetoin	513-86-0	30 ± 2 ^b^	20.2 ± 0.9 ^a^	42 ± 4 ^c^	63 ± 4 ^e^	48 ± 4 ^d^	33 ± 3 ^b^	5
Ethyl Acetate	141-78-6	55 ± 3 ^b^	86.3 ± 0.9 ^d^	29 ± 2 ^a^	75 ± 1 ^c^	89 ± 1 ^d^	86 ± 3 ^d^	4
Ethyl Lactate	97-64-3	22 ± 2 ^c^	12.4 ± 0.5 ^ab^	35 ± 1 ^d^	13.2 ± 0.4 ^b^	49 ± 3 ^e^	9.5 ± 0.2 ^a^	5
Diethyl Succinate	123-25-1	5.0 ± 0.4 ^b^	3.6 ± 0.2 ^a^	8.6 ± 0.4 ^d^	6.6 ± 0.7 ^c^	8.5 ± 0.6 ^d^	5.1 ± 0.5 ^b^	4
2,3-Butanediol (*levo*)	24347-58-8	704 ± 47 ^b^	701 ± 48 ^b^	801 ± 78 ^bc^	831 ± 34 ^c^	1359 ± 129 ^d^	441 ± 21 ^a^	4
2,3-Butanediol (*meso*)	5341-95-7	227 ± 14 ^b^	211 ± 6 ^b^	274 ± 34 ^c^	265 ± 6 ^c^	365 ± 21 ^d^	155 ± 8 ^a^	4
Glycerol (g L^−1^)	56-81-5	7.5 ± 0.7 ^a^	7.0 ± 0.5 ^a^	10 ± 1 ^b^	11 ± 1 ^b^	12 ± 1 ^b^	8.0 ± 0.6 ^a^	2

**Table 3:** Minor volatile compounds (microg L^−1^) quantified in wines form three terroirs during the 2021 and 2022 vintages. ^a–f^ Different letters in the same row indicate homogeneous groups (HG) with statistical differences at the 0.05 significance level. Identification of wines: Aguilar AG2021, AG2022: La Unión LU2021, LU2022: San Acacio SA2021, SA2022. CAS: Chemical Abstracts Service Registry Number.

Compounds	CAS	AG2021	AG2022	LU2021	LU2022	SA2021	SA2022	HG
**Acetates (8)**								
Butyl Acetate	123-86-4	2.71 ± 0.04 ^b^	3.5 ± 0.2 ^d^	2.8 ± 0.2 ^b^	3.7 ± 0.3 ^d^	3.12 ± 0.03 ^c^	2.3 ± 0.2 ^a^	4
Isoamyl Acetate	123-92-2	1989 ± 53 ^a^	4553 ± 240 ^c^	1784 ± 151 ^a^	5999 ± 305 ^d^	3301 ± 292 ^b^	7047 ± 618 ^e^	5
(Z)-3-Hexenyl Acetate	3681-71-8	101 ± 5 ^b^	291 ± 21 ^c^	0.9 ± 0.2 ^a^	5.3 ± 0.8 ^a^	2.32 ± 0.09 ^a^	13.4 ± 0.1 ^a^	3
Hexyl Acetate	142-92-7	44 ± 2 ^b^	128 ± 10 ^c^	21.6 ± 0.3 ^a^	197 ± 6 ^d^	58 ± 5 ^b^	257 ± 22 ^e^	5
Octyl Acetate	112-14-1	3.3 ± 0.2 ^ab^	2.6 ± 0.2 ^a^	4 ± 1 ^c^	3.47 ± 0.03 ^ab^	4.0 ± 0.6 ^c^	6.37 ± 0.00 ^d^	4
Ethyl-phenyl Acetate	101-97-3	1.6 ± 0.1 ^a^	64 ± 2 ^c^	1.0 ± 0.1 ^a^	54 ± 3 ^b^	2.8 ± 0.2 ^a^	90 ± 4 ^d^	4
2-Phenyl-ethyl Acetate	103-45-7	1300 ± 80 ^b^	2394 ± 79 ^d^	760 ± 48 ^a^	2043 ± 87 ^c^	2794 ± 155 ^e^	3361 ± 165 ^f^	6
Geranyl Acetate	105-87-3	23.7 ± 0.9 ^c^	52 ± 5 ^d^	6.5 ± 0.2 ^b^	78 ± 2 ^e^	4.0 ± 0.2 ^ab^	1.80 ± 0.01 ^a^	5
**Ethyl Esters (12)**								
Ethyl Isobutyrate	97-62-1	23.0 ± 0.6 ^c^	13.3 ± 0.5 ^b^	23 ± 2 ^c^	0 ^a^	21 ± 2 ^c^	0 ^a^	3
Ethyl Butyrate	105-54-4	126 ± 6 ^b^	179 ± 13 ^c^	97 ± 4 ^a^	134 ± 4 ^b^	141 ± 13 ^b^	251 ± 7 ^d^	4
Ethyl 2-methyl-butyrate	7452-79-1	4.3 ± 0.4 ^c^	0 ^a^	3.9 ± 0.2 ^b^	0 ^a^	6.3 ± 0.1 ^d^	0 ^a^	4
Ethyl 3-methyl-butyrate	108-64-5	10.0 ± 0.4 ^b^	0 ^a^	9.3 ± 0.5 ^b^	0 ^a^	14 ± 1 ^c^	0 ^a^	3
Ethyl Hexanoate	123-66-0	240 ± 20 ^a^	536 ± 42 ^c^	194 ± 16 ^a^	391 ± 20 ^b^	369 ± 18 ^b^	1552 ± 75 ^d^	4
Ethyl Heptanoate	106-30-9	0.22 ± 0.02 ^b^	0 ^a^	0.22 ± 0.02 ^b^	0 ^a^	0.20 ± 0.01 ^b^	0.50 ± 0.04 ^c^	3
Ethyl Octanoate	106-32-1	281 ± 7 ^b^	633 ± 16 ^d^	131 ± 12 ^a^	591 ± 20 ^c^	666 ± 28 ^e^	2192 ± 19 ^f^	6
Ethyl Decanoate	110-38-3	445 ± 24 ^b^	1178 ± 116 ^d^	74 ± 2 ^a^	1910 ± 45 ^e^	701 ± 41 ^c^	3032 ± 73 ^f^	6
Ethyl Undecanoate	627-90-7	0 ^a^	0 ^a^	0 ^a^	0 ^a^	0 ^a^	1.20 ± 0.01 ^b^	2
Ethyl Dodecanoate	106-33-2	91 ± 6 ^b^	868 ± 45 ^c^	10.4 ± 0.9 ^a^	1271 ± 4 ^d^	23 ± 2 ^a^	1313 ± 19 ^f^	5
Ethyl Tetradecanoate	124-06-1	13 ± 1 ^b^	32 ± 3 ^c^	7 ± 1 ^a^	37 ± 3 ^d^	8.5 ± 0.5 ^ab^	88 ± 5 ^e^	5
Ethyl Hexadecanoate	628-97-7	46 ± 1 ^c^	128 ± 2 ^d^	7 ± 2 ^a^	119 ± 3 ^d^	21.5 ± 0.3 ^b^	159 ± 12 ^e^	5
**Other Esters (5)**								
Phenethyl Butyrate	103-52-6	0 ^a^	1.48 ± 0.02 ^b^	0 ^a^	0 ^a^	1.46 ± 0.08 ^b^	4.4 ± 0.4 ^c^	3
Phenethyl Hexanoate	6290-37-5	0 ^a^	0.68 ± 0.02 ^d^	0 ^a^	0.52 ± 0.02 ^c^	0.31 ± 0.01 ^b^	0.69 ± 0.01 ^d^	4
Hexyl Hexanoate	6378-65-0	0 ^a^	0 ^a^	0 ^a^	0 ^a^	0 ^a^	8.9 ± 0.2 ^b^	2
Phenethyl Benzoate	94-47-3	3.17 ± 0.03 ^a^	3.5 ± 0.1 ^b^	3.1 ± 0.1 ^a^	3.5 ± 0.1 ^b^	3.1 ± 0.2 ^a^	3.46 ± 0.05 ^b^	2
(E)-Methyldihydrojasmonate	2630-39-9	1.5 ± 0.3 ^b^	1.96 ± 0.08 ^c^	1.56 ± 0.05 ^bc^	1.7 ± 0.1 ^bc^	1.3 ± 0.2 ^b^	0.8 ± 0.3 ^a^	3
**Higher Alcohols (6)**								
Hexanol	111-27-3	1564 ± 102 ^b^	1687 ± 67 ^b^	1572 ± 86 ^b^	2304 ± 220 ^c^	1600 ± 6 ^b^	941 ± 23 ^a^	3
2-Ethyl-1-Hexanol	104-76-7	51 ± 1 ^c^	42 ± 6 ^b^	46 ± 3 ^bc^	41 ± 2 ^b^	42 ± 4 ^b^	28 ± 2 ^a^	3
Furanmethanol	98-00-0	0.001 ± 0.000 ^a^	0.001 ± 0.000 ^a^	9.7 ± 0.4 ^c^	4.8 ± 0.1 ^b^	11.7 ± 0.7 ^d^	4.60 ± 0.8 ^b^	4
Octanol	111-87-5	84 ± 13 ^b^	0 ^a^	0 ^a^	172 ± 2 ^c^	173 ± 8 ^c^	230 ± 18 ^d^	4
Decanol	112-30-1	31 ± 1 ^c^	16.3 ± 0.8 ^b^	0 ^a^	45.7 ± 0.9 ^d^	70 ± 3 ^e^	91.0 ± 0.2 ^f^	6
Dodecanol	112-53-8	11 ± 3 ^bc^	8.0 ± 0.6 ^a^	10 ± 1 ^abc^	11.4 ± 0.6 ^c^	12.0 ± 0.9 ^c^	8.8 ± 0.5 ^ab^	3
**Phenols (2)**								
4-Ethyl Guaiacol	2785-89-9	0 ^a^	0 ^a^	0 ^a^	0 ^a^	1944 ± 117 ^b^	0 ^a^	2
2-Methoxy-4-Vinyl-phenol	7786-61-0	42 ± 4 ^ab^	58 ± 6 ^b^	28 ± 3 ^a^	81 ± 1 ^c^	42 ± 3 ^ab^	338 ± 22 ^d^	4
**Lactones (4)**								
γ-Butyrolactone	96-48-0	13,367 ± 162 ^a^	15,996 ± 745 ^b^	21,014 ± 1767 ^c^	16,077 ± 885 ^b^	16,286 ± 864 ^b^	12,117 ± 1022 ^a^	3
γ-Nonalactone	104-61-0	34 ± 2 ^e^	21.9 ± 0.9 ^d^	18.1 ± 0.2 ^bc^	20 ± 2 ^cd^	17.2 ± 0.1 b	8.57 ± 0.07 a	5
γ-Crotonolactone	497-23-4	0.001 ± 0.000 ^b^	0 ^a^	0.001 ± 0.000 ^b^	0 ^a^	0.001 ± 0.000 ^b^	0.001 ± 0.000	2
β-Damascenone	23726-93-4	16.4 ± 0.6 ^b^	37 ± 4 ^d^	3.8 ± 0.2 ^a^	56 ± 1 e	23 ± 1 ^c^	88 ± 2 ^f^	6
**Carbonyl Compounds (8)**								
Hexanal	66-25-1	5.7 ± 0.4 ^c^	6.0 ± 0.7 ^bc^	4.7 ± 0.2 ^a^	4.95 ± 0.07 ^ab^	5.9 ± 0.3 ^c^	6.8 ± 0.7 ^d^	4
Furfural	98-01-1	366 ± 11 ^b^	0.001 ± 0.000 ^a^	1009 ± 99 ^d^	426 ± 28 ^b^	660 ± 69 ^c^	388 ± 14 ^b^	4
Benzaldehyde	100-52-7	0 ^a^	0 ^a^	0 ^a^	0 ^a^	1.7 ± 0.2 ^b^	2.87 ± 0.03 ^c^	3
Octanal	124-13-0	0 ^a^	0.001 ± 0.000 ^a^	1.6 ± 0.1 ^a^	116 ± 9 ^b^	1.46 ± 0.02 ^a^	1.85 ± 0.06 ^a^	2
Decanal	112-31-2	6.6 ± 0.5 ^bc^	5.2 ± 0.2 ^a^	6.15 ± 0.05 ^b^	7.4 ± 0.2 ^c^	9 ± 1 ^d^	9.4 ± 0.3 ^d^	4
(E)-2-Octenal	2548-87-0	0 ^a^	0 ^a^	0 ^a^	0 ^a^	0 ^a^	6.9 ± 0.3 ^b^	2
(E)-2-Nonenal	18829-56-6	0 ^a^	0 ^a^	0 ^a^	0 ^a^	4.3 ± 0.3 ^b^	9.9 ± 0.8 ^c^	3
3-Heptanone	106-35-4	0.001 ± 0.000 ^a^	1.1 ± 0.2 ^b^	0.001 ± 0.000 ^a^	1.2 ± 0.1 ^b^	0.001 ± 0.000 ^a^	0.001 ± 0.000 ^a^	2
**Terpenes and derivates (6)**								
(DL)-Limonene	138-86-3	0.001 ± 0.000 ^a^	0 ^a^	0.001 ± 0.000 ^a^	0.001 ± 0.000 ^a^	0.001 ± 0.000 ^a^	4049 ± 233 ^b^	2
(E)-Geranyl Acetone	3796-70-1	1.0 ± 0.2 ^a^	1.4 ± 0.1 ^b^	2.2 ± 0.1 ^d^	1.90 ± 0.01 ^c^	1.26 ± 0.07 ^b^	0.87 ± 0.06 ^a^	4
(Z)-Geranyl Acetone	3879-26-3	1.90 ± 0.05 ^a^	1.88 ± 0.03 ^a^	1.91 ± 0.01 ^a^	2.3 ± 0.3 ^c^	2.0 ± 0.1 ^ab^	2.2 ± 0.2 ^bc^	3
(E)-Citral	141-27-5	15 ± 1 ^b^	0 ^a^	0 ^a^	21.5 ± 0.1 ^c^	26 ± 3 ^d^	38 ± 3 ^e^	5
(Z)-Nerolidol	7212-44-4	0.02 ± 0.00 ^a^	0.001 ± 0.000 ^a^	0.001 ± 0.000 ^a^	0.15 ± 0.04 ^c^	0.07 ± 0.02 ^b^	0.001 ± 0.000 ^a^	3
Farnesol	4602-84-0	0.001 ± 0.000 ^a^	0.001 ± 0.000 ^a^	0.001 ± 0.000 ^a^	1.2 ± 0.5 ^b^	0.001 ± 0.000 ^a^	0.001 ± 0.000 ^a^	2
**Miscellaneous (1)**								
2-Pentylfuran	3777-69-3	0.001 ± 0.000 ^a^	0.001 ± 0.000 ^a^	0.001 ± 0.000 ^a^	0.001 ± 0.000 ^a^	0.001 ± 0.000 ^a^	12.4 ± 0.4 ^b^	2

## Data Availability

Data is contained within the article or Supplementary Material.

## Supplementary Materials

The following supporting information can be downloaded at: https://www.mdpi.com/article/10.3390/molecules30020238/s1, Table S1: Multiple Analysis of Variance (MANOVA) with terroir, vintage and interaction as variation factors. Table S2: Loads of the major volatile compounds in the first three Principal components; Table S3: Loads of the 52 minor volatile compounds in Principal components.
