# Supplementary material for: Analytical Differentiation of Wines from Three Terroirs Located in a Warm Winegrowing Area Based on Their Volatilome
Source: Molecules. 2025 Jan 9;30(2):238. doi: 10.3390/molecules30020238 (PMC11768053; doi:10.3390/molecules30020238)
Supplement: Supplementary file 1 [file molecules-30-00238-s001.zip › molecules-3400166-supplementary.pdf]

Supplementary Material

Analytical Differentiation of Wines from Three Terroirs Located in a Warm Winegrowing Area Based on Their Volatilome

Table S1. Multiple Analysis of Variance (MANOVA) with terroir, vintage and interaction as variation factors.

Parameters or Compounds	*Terroir*	*Vintage*	*Interaction*
General parameters			
pH	0.0000	1.0000	0.0000
Total Acidity	0.0000	0.1089	0.0000
Density	0.0000	1.0000	1.0000
Reducing sugars	0.3664	0.0001	0.0074
Absorbance 620 nm	0.0000	0.4369	0.0000
Major volatiles			
2-Methyl-1-butanol	0.0000	0.0000	0.0891
Acetoin	0.0000	0.3248	0.0000
Minor volatiles			
Octyl acetate	0.0000	0.1895	0.0003
Phenethyl benzoate	0.8313	0.0001	0.7609
(E)-Methyldihydrojasmonate	0.0004	1.0000	0.0110
Hexanol	0.0000	0.2266	0.0000
2-Ethyl-1-hexanol	0.0003	0.0001	0.1343
Hexanal	0.0002	0.0371	0.4614
Decanal	0.0000	0.7702	0.0026
(E)-Geranyl cetone	0.0000	0.1751	0.0000

p-values of the compounds affected not significantly by the two factors and their interaction. All remaining compounds quantified are dependent of terroir, vintage and their interaction. *p*-values ≤ 0.05 are statistically significant.

Table S2. Loads of the major volatile compounds in the first three Principal components.

	Component 1	*Component 2*	*Component 3*
Variance explained	48,45 %	24,67 %	19,66 %
Cumulative variance	48,45 %	73.12 %	92,78 %
Acetaldehyde	**0,274149**	-0,0100892	-0,381384
Ethyl acetate	-0,0997195	**-0,206758**	0,469443
1,1-Diethoxyethane	0,171809	**0,230945**	-0,424986
Methanol	**0,29864**	**0,209674**	-0,189618
1-Propanol	0,242047	**-0,213212**	0,331429
Isobutanol	0,0571141	**0,489621**	0,0981173
2-Methyl-1-butanol	0,0972469	**0,452344**	0,228024
3-Methyl-1-butanol	0,207677	**0,313807**	0,295899
Acetoin	**0,270323**	0,192426	0,269596
Ethyl lactate	**0,308539**	**-0,245427**	-0,148264
2,3-Butanediol (levo)	**0,296141**	**-0,26503**	0,132127
2,3-Butanediol (meso)	**0,328711**	-0,187991	0,0695944
Diethyl succinate	**0,355419**	0,0338827	-0,0779837
2-Phenylethanol	**0,286825**	**-0,253524**	-0,0378767
Glycerol	**0,330374**	0,0575054	0,184653

This table shows the contribution of each variable to the equations of the principal components. For example, the first principal component has the equation

PC1= 0,274149*Acetaldehyde - 0,0997195*Ethyl acetate + 0,171809*1,1-Diethoxyethane + 0,29864*Methanol + 0,242047*1-Propanol + 0,0571141*Isobutanol + 0,0972469*2-Methyl-1-butanol + 0,207677*3-Methyl-1-butanol + 0,270323*Acetoin + 0,308539*Ethyl lactate + 0,296141*2,3-Butanediol (levo) + 0,328711*2,3-Butanediol (meso) + 0,355419*Diethyl succinate + 0,286825*2-Phenyl-ethanol + 0,330374*Glycerol

Table S3. Loads of the 52 minor volatile compounds in Principal components.

	Component 1	*Component 2*	*Component 3*
Variance explained	51.28 %	22.75 %	11.58 %
Cumulative variance	51.28 %	74.03 %	85.61 %
Ethyl Isobutyrate	0,151371	-0,165635	-0,0997396
Ethyl Butanoate	**-0,186014**	0,00871536	-0,105182
Hexanal	-0,13732	-0,0636065	-0,114669
Butyl Acetate	0,0758763	**0,228222**	0,100496
Furfural	0,0660542	-0,171274	0,210894
Ethyl 2-methylbutanoate	0,112454	**-0,216738**	0,0983828
Ethyl 3-methylbutanoate	0,114751	**-0,216425**	0,091857
2-Furanmethanol	0,0234466	-0,150084	0,285305
Hexanol	0,110322	**0,204413**	0,176688
3-Heptanone	0,00979515	**0,284742**	0,0039286
Isoamyl Acetate	-0,169055	0,137215	0,0759493
Î³-Butyrolactone	0,127774	-0,00846398	0,076406
Ethyl Hexanoate	**-0,194396**	-0,0256984	-0,052259
Benzaldehyde	-0,161553	-0,143006	0,0804102
Pentylfuran	**-0,18632**	-0,0726152	-0,0346049
Octanal	0,00252247	**0,238987**	0,213897
Z-3-hexen-1-ol Acetate	0,0255904	0,126219	-0,336552
Hexyl Acetate	-0,174281	0,131152	0,0464459
2-Ethyl-1-hexanol	**0,173323**	-0,0191125	-0,0465352
Limonene	**-0,186154**	-0,0723769	-0,0356406
E-2-Octenal	**-0,186299**	-0,0726045	-0,0344709
Octanol	-0,138151	-0,0311417	0,250492
Ethyl Heptanoate	-0,120873	**-0,214455**	-0,0201335
E-2-Nonenal	-0,173637	-0,129051	0,0525729
Ethyl Octanoate	**-0,195945**	-0,0318844	-0,00487647
Decanal	-0,122324	-0,123564	0,2477
Octyl Acetate	-0,145063	-0,135589	0,0832058
Ethyl Phenylacetate	-0,164542	0,147559	-0,0732087
2-Phenylethyl Acetate	-0,160112	0,0102363	0,0481652
2-Methoxy-4-vinylphenol	**-0,192516**	-0,0319349	-0,0178738
Geranyl Acetate	0,0320357	**0,285292**	0,0358612
Decanol	-0,156299	-0,0662283	0,165994
4-Ethylguaiacol	0,0229347	-0,133428	0,205031
Î³-Nonalactone	0,137216	0,0355673	-0,111329
Î²-Damascenone	**-0,185998**	0,0908004	0,0415896
Hexyl Hexanoate	**-0,186355**	-0,0726042	-0,0346386
Ethyl Decanoate	**-0,18615**	0,0886886	0,0492238
E-Geranyl Acetone	0,106053	0,0943103	0,129591
Phenethyl Butyrate	**-0,181558**	-0,0710464	-0,0663501
Z-Geranyl Acetone	-0,102991	0,0903953	0,259605
Dodecanol	0,0612962	-0,041633	0,280605
Ethyl Undecanoate	**-0,186394**	-0,0725861	-0,0349057
Ethyl Dodecanoate	-0,147172	**0,192949**	0,0176187
Phenethyl Hexanoate	-0,143899	0,154944	-0,0269094
E-Methyldihydrojasmonate	0,129593	0,159956	-0,0950127
Z-Nerolidol	0,0202041	0,154551	0,329743
Farnesol	0,00133371	**0,206687**	0,255234
Ethyl Tetradecanoate	**-0,191407**	0,0473027	-0,0369461
Phenethyl Benzoate	-0,102288	**0,200778**	-0,0263929
Ethyl Hexadecanoate	-0,155368	0,170032	-0,0855831

This table shows the contribution of each variable to the equations of the principal components. The values of the variables in the equation have been standardized by subtracting their means and dividing by their standard deviations.
